# Proteomic Analysis of Differentially Expressed Proteins in *Fenneropenaeus chinensis* Hemocytes upon White Spot Syndrome Virus Infection

**DOI:** 10.1371/journal.pone.0089962

**Published:** 2014-02-27

**Authors:** Wei Li, Xiaoqian Tang, Jing Xing, Xiuzhen Sheng, Wenbin Zhan

**Affiliations:** Laboratory of Pathology and Immunology of Aquatic Animals, Ocean University of China, Qingdao, Shandong, China; Institute of Oceanology, Chinese Academy of Sciences, China

## Abstract

To elucidate molecular responses of shrimp hemocytes to white spot syndrome virus (WSSV) infection, two-dimensional gel electrophoresis was applied to investigate differentially expressed proteins in hemocytes of Chinese shrimp (*Fenneropenaeus chinensis*) at 24 h post infection (hpi). Approximately 580 protein spots were detected in hemocytes of healthy and WSSV-infected shrimps. Quantitative intensity analysis revealed 26 protein spots were significantly up-regulated, and 19 spots were significantly down-regulated. By mass spectrometry, small ubiquitin-like modifier (SUMO) 1, cytosolic MnSOD, triosephosphate isomerase, tubulin alpha-1 chain, microtubule-actin cross-linking factor 1, nuclear receptor E75 protein, vacuolar ATP synthase subunit B L form, inositol 1,4,5-trisphosphate receptor, arginine kinase, etc., amounting to 33 differentially modulated proteins were identified successfully. According to Gene Ontology annotation, the identified proteins were classified into nine categories, consisting of immune related proteins, stimulus response proteins, proteins involved in glucose metabolic process, cytoskeleton proteins, DNA or protein binding proteins, proteins involved in steroid hormone mediated signal pathway, ATP synthases, proteins involved in transmembrane transport and ungrouped proteins. Meanwhile, the expression profiles of three up-regulated proteins (SUMO, heat shock protein 70, and arginine kinase) and one down-regulated protein (prophenoloxidase) were further analyzed by real-time RT-PCR at the transcription level after WSSV infection. The results showed that SUMO and heat shock protein 70 were significantly up-regulated at each sampling time point, while arginine kinase was significantly up-regulated at 12 and 24 hpi. In contrast, prophenoloxidase was significantly down-regulated at each sampling time point. The results of this work provided preliminary data on proteins in shrimp hemocytes involved in WSSV infection.

## Introduction

White spot syndrome virus (WSSV) is one of the most important pathogens in shrimp, and can cause considerable economic losses to shrimp culture industry worldwide [1]. Over the past two decades, significant progresses have been made on the epidemiology, pathology and characterization of WSSV, but no effective treatment was available to control the occurrence and spread of the disease [2]. In recent years, considerable efforts were focused toward WSSV infection mechanism and obtaining substantial information on the host cellular and viral molecules involved in WSSV infection [3]–[7], which had important implications for developing effective therapeutic and preventive measures against white spot disease, however, these information was still limited.

Viral infections would induce cellular antiviral responses, and the infectious virus would also try to manipulate a variety of physiological processes in host cells for facilitating their successful replication [8]. In this process, numerous cellular molecules involved in virus infection would be induced or suppressed. Thus, identification of differentially expressed genes or proteins during viral infection has great significance for gaining insights into the WSSV infection mechanism and host antiviral response. Two-dimensional gel electrophoresis (2-DE)-mass spectrometry (MS) is the most widely used technique of proteomics [9]. Recently, this technique has been employed for the comparative analysis of altered proteins of shrimp upon viral disease, and dozens of proteins involved in viral infection have been identified [10]–[14]. However, up to now, the information on molecular response in shrimp hemocytes against WSSV infection is still limited.

It is well known that hemocytes play a key role in immune response in shrimp [15]. Therefore, in the present work, 2DE-MS approach was used to identify altered expressed proteins in hemocytes of Chinese shrimp (*Fenneropenaeus chinensis*) after WSSV infection, and real-time RT-PCR was applied to investigate the expression dynamics of some altered proteins at mRNA level.

## Materials and Methods

### Shrimp

Nature-captured shrimps (*F. chinensis*) with average size of 15–17 cm in length were obtained from a local market in Qingdao, Shandong province, China. The shrimps were randomly selected for detection of WSSV by PCR to ensure that they were WSSV-free before the experiment. The shrimps were maintained in aerated seawater at 30‰ salinity, and half of the water was refreshed every day.

### Preparation of Virus Inoculum

The WSSV inoculum used in this work was prepared using the gills of naturally WSSV-infected *F. chinensis* and quantified according to the previous methods [16], [17]. Briefly, the gill tissue was homogenized in sterile phosphate buffered saline (PBS, pH 7.4) for 5 min at a ratio of 10% w/v using a mortar and pestle, and then the homogenate was centrifuged at 600 g for 20 min at 4°C. The supernatant was passed through a 450 nm membrane filter. Subsequently, the quantification of the virus was performed, and the virus inoculum was stored at −80°C until used.

### Virus Challenge

After acclimatization at 25°C for 5 days, the shrimps were averagely divided into two groups, with each group containing 60 shrimps. The shrimps of WSSV-infected group were injected with 50 µl (10^7^ copies) of diluted virus inoculum through the lateral surface of the fourth abdominal segment. The shrimps in control group were injected with PBS instead of WSSV inoculum. After injection, the disease symptoms and mortality of inoculated shrimps were recorded. The hemolymph was randomly collected from 6 shrimps in each group at 0, 6, 12, 24, 36 and 48 h post-infection (hpi) for WSSV detection by nested PCR according to the Manual of Diagnostic Tests for Aquatic Animals (http://www.oie.int/international-standard-setting/aquatic-manual/access-online/). Meanwhile, the hemocytes in each hemolymph sample were isolated according to the method described previously [16], and suspended in TRIzol^®^ reagent (Invitrogen, USA) for total RNA extraction.

### Preparation of Hemocyte Proteins

12 shrimps were randomly sampled from each group at at 24 hpi, the late stage of WSSV replication cycle [18]. The hemocytes were isolated and suspended in lysis buffer (7 M urea, 2 M thiourea, 4% CHAPS, 40 mM Tris-base, 65 mM DTT, and 1% protease inhibitor cocktail) for 2 h incubation at 4°C with gentle shaking. Then the hemocytes lysate was sonicated under the pulse mode (2 s on, 5 s off, 80 W) for 20 s. After sonication, the cell lysate was centrifuged at 16 000 g at 4°C for 40 min, and the supernatant was collected. The protein concentration of supernatant was determined by Bradford assay [19] and adjusted to 2.4 mg/ml with rehydration buffer (8 M urea, 4% w/v CHAPS, 30 mM DTT and 0.5% IPG buffer (GE Healthcare) for later 2-DE analysis.

### 2-DE Analysis

Hemocyte proteins form 4 shrimps were mixed as a pool, and three independent pools from each group (12 shrimps per group) were separated by 2-DE using the Ettan IPGphor II system (Amersham Biosciences). 250 µl rehydration buffer containing 600 µg hemocyte proteins of WSSV-infected or healthy shrimps was loaded onto each immobilized pH gradient (IPG) strip (Immobiline TM DryStrip, non-linear pH 3–10, 13 cm, GE Healthcare), and the rehydration was performed at 50 mA per strip for 12 h. The IEF was carried out at 20°C using a continuous increase voltage (up to 8000 V) to reach 50 000 Vh. Prior to the second dimension, the focused IPG strips were equilibrated in reducing solution (6 M Urea, 30% glycerol, 2% SDS, 50 mM Tris-HCl, 0.002% bromophenol blue, 1% DTT, pH 8.8) and alkylating solution (6 M Urea, 30% glycerol, 2% SDS, 50 mM Tris-HCl, 0.002% bromophenol blue, 2.5% iodoacetamide, pH 8.8) each for 15 min at room temperature. Then the strips were sealed by 0.5% melted agarose and run in 12.5% SDS-PAGE for the second dimension electrophoresis, and the gels were stained by Coomassie brilliant blue (CBB) G-250.

### Image Analysis

The gels were scanned using a Typhoon 9400 scanner (Amersham Biosciences), and the gel analysis was performed using PDQuest software (Bio-Rad). Comparative analysis of protein spots was performed by matching corresponding spots across different gels (WSSV-infected and control group). Spot volume was normalized with the intensity volume of each spot to the total intensity volume of all spots detected on the gel, and subjected to statistical analysis with Student’s *t*-test to compare the normalized intensity volumes of individual spots of the control group to those of the infected group. *P* values less than 0.05 were considered statistically significant. Only differentially expressed proteins (≥1.5-fold) were excised and subjected to subsequent identification by MS.

### MS Analysis and Gene Ontology (GO) Annotation

The differentially expressed protein spots were excised from 2D gels and subjected to in-gel trypsin digestion, and followed by MALDI-TOF-MS/MS analysis as described by Bu et al. [20]. The MS and MS/MS data were first searched against the NCBI protein and expressed sequence tag (EST) database performed by MASCOT (Version 2.1.03, Matrix Science, London, UK). For unidentified protein spots, two local databases were searched additionally, one was Decapoda Protein Database (20508 sequences, 4416699 residues) provided by MS Laboratory (Beijing Protein Innovation Co., Ltd, China). Another database was constructed by combining the EST sequences (total 211084 sequences) of three shrimp species, including *F. chinensis* (10446 sequences), *Penaeus monodon* (39397 sequences), and *Litopenaeus vannamei* (16241 sequences), the EST data were all downloaded from NCBI (September, 2012). The search parameters were set as follows: up to one missed cleavage was allowed, trypsin digest, carbamidomethyl (C) was fixed modification, Oxidation (M) was variable modification, 100 ppm as peptide mass tolerance and 0.3 Da as MS/MS tolerance. Mascot score which was over 50 was considered as a positive hit. The identified proteins were classified by their GO annotations of biological processes and molecular functions using AmiGO against the GO database (http://amigo.geneontology.org/cgi-bin/amigo/go.cgi).

### Confirmation of the Proteomic Data by Real-time RT-PCR

Total RNA was extracted from the shrimp hemocytes prepared above using TRIzol^®^ reagent. 2 µg total RNA was used to the first-strand cDNA synthesis by random primers and M-MLV reverse transcriptase (Takara, Japan). Quantitative real-time RT-PCR was carried out using SYBR Premix Ex Taq™ (Takara, Japan) in a Thermal Cycler Dice^®^ Real Time System (Eppendorf, Germany) with the following conditions: 95°C for 2 min, followed by 40 cycles of 95°C for 10 s, 58°C for 10 s, and 72°C for 20 s. A dissociation curve with a single peak was used to monitoring the amplified product. 18S rRNA was used as the internal control. The primer sequences for the genes of four identified proteins and 18S rRNA were listed in Table 1. The data were calculated according to 2^−ΔΔCt^ method and statistically analyzed by SPSS 19.0.

**Table 1:** Primers used in real-time RT-PCR analysis.

Target gene (accession no.)	Primer name	Primer sequence (5′-3′)
SUMO (KF773849)	SUMO-F	CAGAAGGGGAAGGGAACGA
	SUMO-R	AACGCAGCGATGCTACAGG
heat shock protein 70 (FJ167398)	HSP70-F	ACAAGCAGAGCGTAGGAAAGGC
	HSP70-R	TCGTTCACAGGCAGTTCGGAGA
arginine kinase (AY661542)	AK-F	TCGGTGATGTTACCTCCTTCG
	AK-R	GCTTCTGCTGGACTTCCTTGC
Prophenoloxidase (EU015060)	proPO-F	TGGGTTTTGACCGTGACAGG
	proPO-R	ACCGTCCTTGATGGGAATGC
18S rRNA (AY438005)	18S-F	ACAATGGCTATCACGGGTAACG
	18S-R	CTGCTGCCTTCCTTAGATGTGGTA

## Results

### WSSV Infection in *F. chinensis*

**Figure 1:**
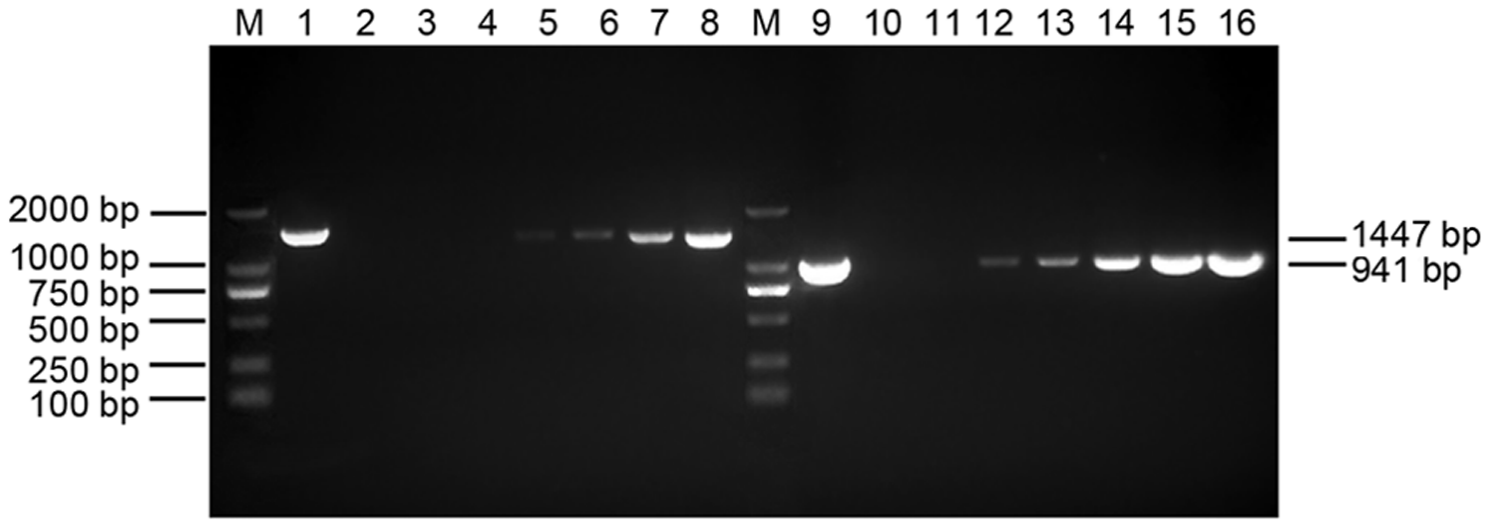
Detection of WSSV in hemocytes of *F. chinensis* post WSSV infection. Lane M, marker; Lane 1–2, the first-step PCR products of positive control and negative control; Lane 3–8, the first-step PCR products of hemocytes at 0, 6, 12, 24, 36 and 48 hpi; Lane 9–10, the second-step PCR products of positive control and negative control; Lane 11–16, the second-step PCR products of hemocytes at 0, 6, 12, 24, 36 and 48 hpi.

After injection with WSSV inoculum, shrimps displayed signs of reduction in food consumption, red discoloration of body, especially on pleopod, and lethargy occured after 3–4 days post infection. The first-step PCR showed that an expected band with 1447 bp was first detected at 12 hpi, and a 941 bp positive product could be first observed at 6 hpi by nested PCR. The intensity of the PCR product bands increased as time extended (Fig. 1). However, the shrimps injected with PBS were detected to be WSSV-negative by PCR (figure not shown).

### Differentially Expressed Proteins in WSSV-infected Hemocytes

**Figure 2:**
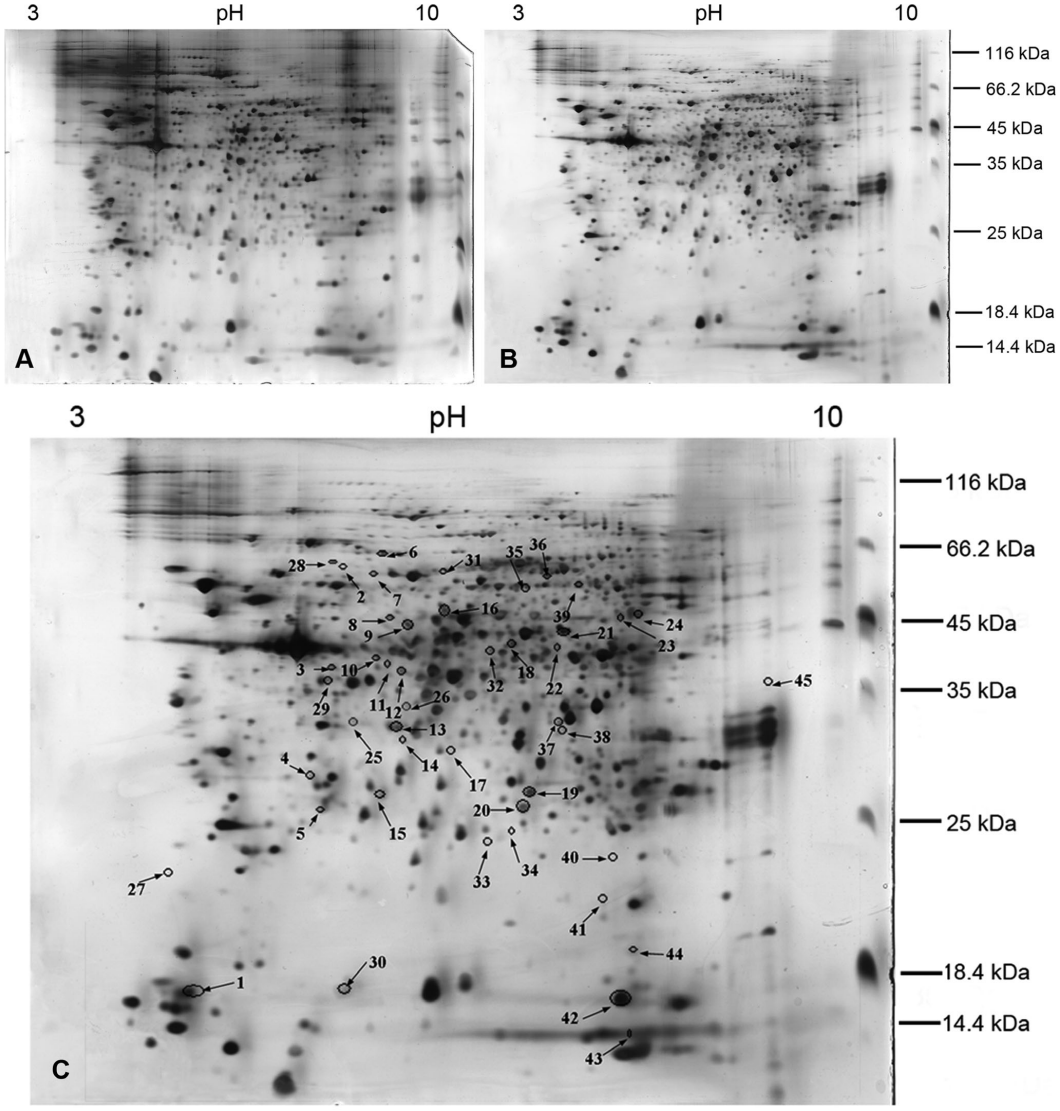
Representative 2D gel maps of hemocytes proteins of *F. chinensis* at 24 hpi. (A) Control hemocytes, (B) WSSV-infected hemocytes, (C) Differentially expressed proteins in a 2D gel. The horizontal axis of the gels was the isoelectric focusing dimension, which stretched in the range of pH 3–10 in a non-linear fashion; the vertical axis was the polyacrylamide gel (12.5%) dimension. Spots were visualized by CBB-G250 staining. Differentially expressed protein spots were circled and labeled with numbers in Fig. 2. C, which correspond to the numbers present in Table S1.

The hemocyte proteins extracted from WSSV-infected and control shrimps were separated by 2-DE, and approximately 580 protein spots were detected by PDQuest software on each gel. Most of these protein spots had p*I*s within the range of 5–8 and had the molecular weights (MWs) ranged from 25 to 60 kDa (Fig. 2). Comparative analysis of the control (Fig. 2A) and infected (Fig. 2B) hemocyte proteomes at 24 hpi was performed. A total of 45 protein spots displayed significant expression changes between the two groups, including 26 up-regulated spots and 19 down-regulated spots. The differentially expressed protein spots were marked in Fig. 2C.

### Protein Identification and GO Annotation

**Figure 3:**
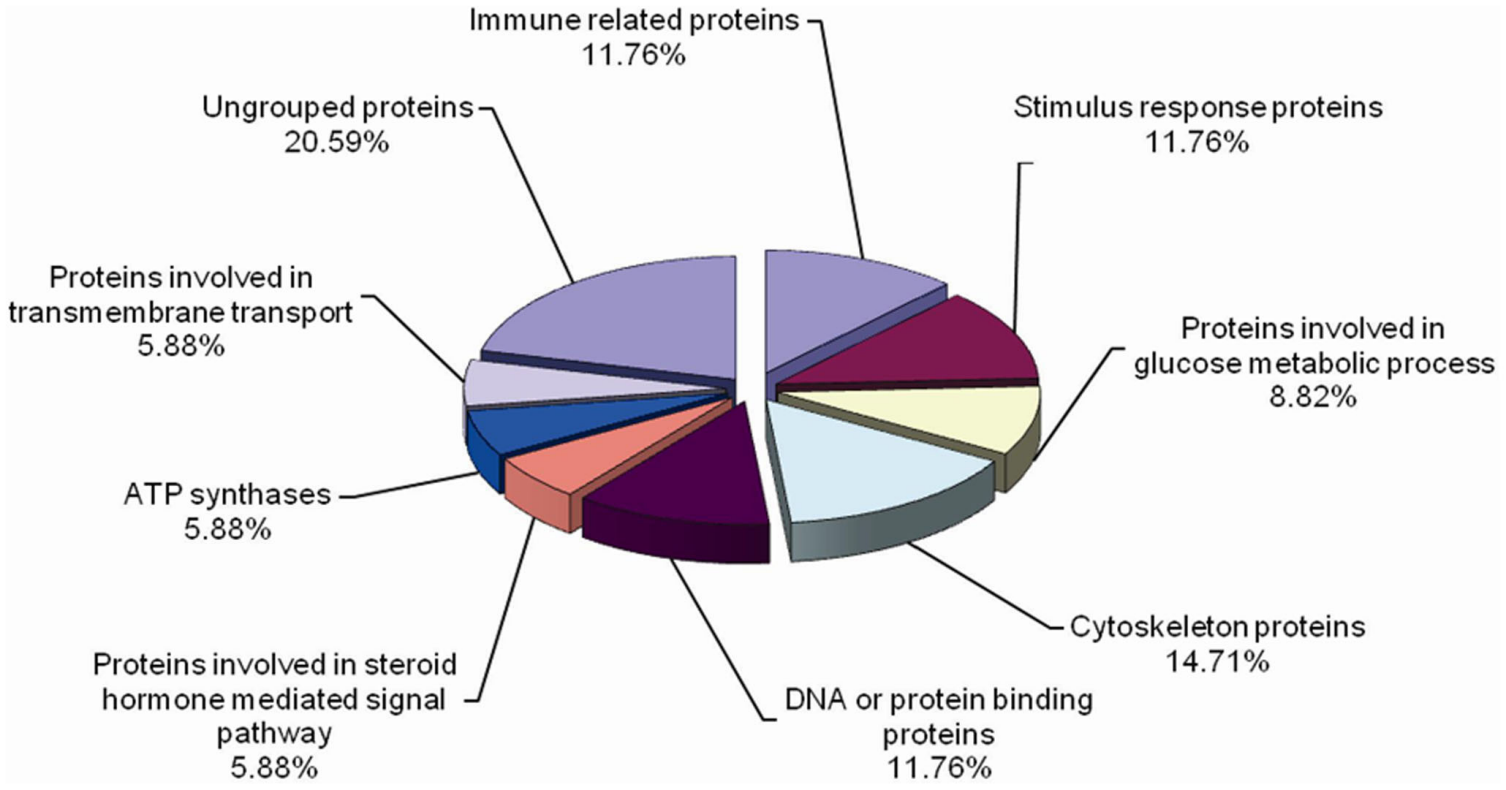
Functional classification of proteins identified in hemocytes of *F. chinensis*. A total of 33 identified proteins were categorized into nine groups, and the percentage contribution of each category was calculated.

A total of 45 up- or down-regulated protein spots were excised and subjected to MS analysis. 33 proteins were successfully identified from 34 protein spots, for spot 11 and 12 were identified to be the same protein. The identified differentially expressed hemocyte proteins could be classified into nine categories based on their GO annotations. The detailed information of identified proteins was summarized in Table S1, and the percentage contribution of each category was shown in Fig. 3.

The nine categories included immune related proteins, stimulus response proteins, proteins involved in glucose metabolic process, cytoskeleton proteins, DNA or protein binding proteins, proteins involved in steroid hormone mediated signal pathway, ATP synthases, proteins involved in transmembrane transport and ungrouped proteins.

### Real-time RT-PCR

**Figure 4:**
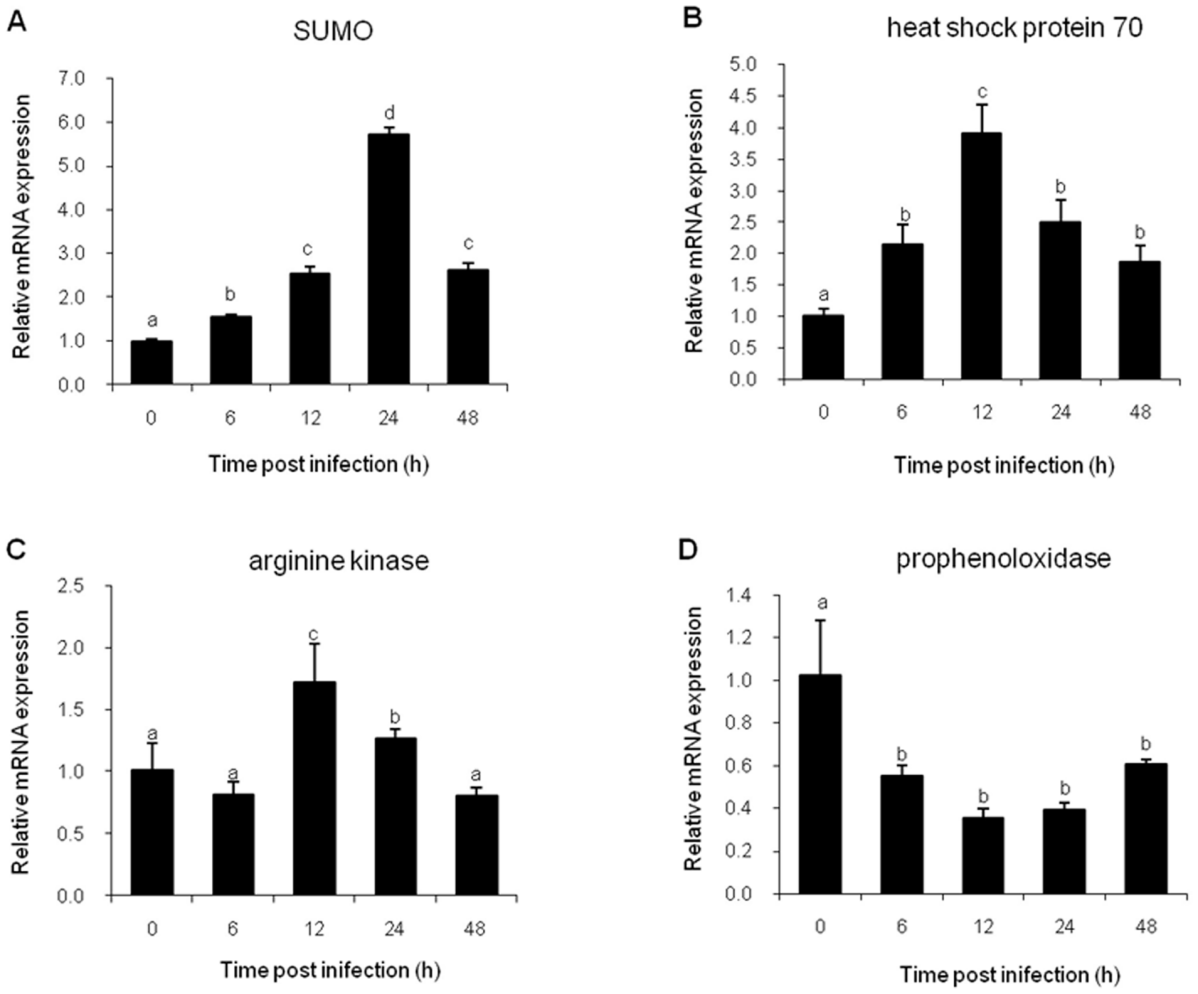
Expression profiles of four genes in hemocytes of *F. chinensis* post WSSV infection. The 18S rRNA was used for normalization of PCR reactions. The bar graphs represent the relative fold changes at each time point post infection, together with error bars which represent mean ± standard deviation (n = 3). *Different letters* indicates significant difference between groups (*p*<0.05). (A) SUMO, (B) heat shock protein 70, (C) arginine kinase, (D) prophenoloxidase.

Three up-regulated proteins (SUMO, heat shock protein 70, and arginine kinase) and one down-regulated protein (prophenoloxidase) were selected for further analysis in their mRNA levels by quantitative real-time PCR. The expression profiles of these genes post WSSV infection were shown in Fig. 4. The expression levels of SUMO and heat shock protein 70 were significantly up-regulated post WSSV infection and reached their peak levels at 24 hpi and 12 hpi respectively. (Fig. 4A, Fig. 4B). The arginine kinase transcript level was detected to be significantly higher than the control level at 12 and 24 hpi (Fig. 4C). In contrast, the prophenoloxidase transcription level decreased significantly post WSSV infection and displayed a lowest level at 12 hpi (Fig. 4D). The up- or down-regulated tendency of the four selected genes at transcription levels obtained from real-time PCR was consistent with the 2-DE data.

## Discussion

To date, proteome responses of organisms to disease have been widely studied [21], [22], however, the knowledge on proteins involved in crustacean responses to viral pathogen infection was still limited. In the present work, 2DE-MS technology was used to explore the molecular responses of *F. chinensis* during WSSV infection. Proteomic analysis was performed using the hemocytes collected at 24 hpi based on the consideration of the successful identification of differentially expressed proteins. For previous work had shown WSSV replication cycle *in vivo* to be approximate 24 hpi, and a large number of hemocytes were infected at the stage [18]. Under this condition, a great many hemocyte proteins participate in shrimp antiviral responses, which is helpful to obtain more information on altered proteins involved in WSSV infection. The results revealed 45 protein spots in the 2DE gels of shrimp hemocytes had significant alterations in their expression levels upon WSSV infection. Among the 45 protein spots, 34 proteins spots were successfully identified by MS analysis. However, 11 spots were not identified successfully by searching against the NCBI non-redundant database and the local databases, which suggested that protein sequence information and EST data for shrimp are still not rich.

Four immune related proteins were identified in the present work. SUMO-1 is an ubiquitin-related protein that participates in multiple biological processes, and plays a vital role in transcriptional regulation [23]. In recent years, growing numbers of viral proteins have been found to conjugate with SUMO-1 [24], [25]. More recently, the modification of WSSV IE proteins by SUMOylation in crayfish was reported and the SUMOylation facilitated reproduction of WSSV, and the expression of SUMO gene in crayfish was also detected to be up-regulated post WSSV infection [26], which was consistent with our present result. These results suggest that SUMOylation may play an important role in WSSV replication in shrimp. RNA helicase serves multiple roles at the virus-host interface. In some situations, RNA helicase is an essential host factor to promote viral replication; however, in other cases it serves as a cellular sensor to trigger the antiviral state in response to viral infection [27]. Up to date, however, the function of RNA helicase in shrimp innate immunity remains unknown. In this investigation, it was found that RNA helicase was significantly up-regulated in virus-infected shrimp, suggesting that it was involved in shrimp immunity. Dynein is a motor protein in cells. It has been reported that many viruses are transported along microtubules through the cellular dynein components in vertebrate [28]. In the present work, an up-regulated expression of cytoplasmic dynein 2 heavy chain 1 was detected in WSSV-infected hemocytes, which might promote the transportation process of WSSV in infected cells. Caspase, a central effector in apoptosis [29] was also identified. We speculate that caspase participates in shrimp apoptosis caused by viral infection.

Alterations of stimulus response proteins were observed. MnSOD is a member of the SOD family, playing an important role in elimination of reactive oxygen species (ROS) to protect cells from damage caused by reactive molecules accumulation [30]. The up-regulation of cytosolic MnSOD in the infected hemocytes may be essential in reducing ROS and the oxidative stress caused by WSSV infection. Phosphatase 2, a key enzyme in abscisic acid response, belongs to serine/threonine protein phosphatase family [31]. And a protein of the family in *L. vannamei* has been implicated in the latent-lytic life cycle of WSSV through interacting with WSSV427 [32]. Heat shock protein 70 plays a role in the stress response by acting as a molecular chaperone. The up-regulation of the protein induced by WSSV infection was also observed in mud crab hemolymph [33]. Alcohol dehydrogenase is a typical hypoxic response protein [34], while the function of it in WSSV infection is still unclear and needs to be further investigated.

Five cytoskeletal proteins and four DNA or protein binding proteins were identified. Among them, two proteins of tubulin family (tubulin alpha-1 chain and tubulin beta-1 chain), microtubule-actin cross-linking factor 1 and CAP-Gly domain-containing linker protein 2 are microtubule-related proteins. The expression levels of the four proteins were up-regulated post WSSV infection. All these data suggest that microtubule-related proteins may form a network to participate in WSSV infection. In addition, three cytoskeletal proteins belonged to actin family were indentified. In previous work, β-actin was shown to bind to WSSV nucleocapsid protein VP26 in *F. chinensis* [35], and the binding was also observed in *Procambarus clarkii* [36]. We speculate that actin plays an important role in WSSV infection process. HMGBb and Histone H2B were two other DNA or protein binding proteins, and their expression levels were down-regulated post WSSV infection. HMGBb is a protein belonging to HMGB family. Recently, HMGB was reported to be involved in WSSV gene regulation by interacting with transcription factors STAT and Dorsal in *L. vannmei* [37]. Histone H2B is a nuclear histone protein. And a recent report shows that the highly expressed WSSV protein, ICP11, can disrupt shrimp nucleosome assembly by binding to a group of histone, including H2A, H2B, H3 and H2A.x [38].

Three proteins involved in glucose metabolic process (triosephosphate isomerase, phosphopyruvate hydratase and glucose-6- phosphate isomerase) and two subunits of ATP synthase were identified in the present work. Among them, triosephosphate isomerase and phosphopyruvate hydratase have also been identified in hepatopancreas of *F. chinensis* post WSSV infection [14]. ATP synthase is a key enzyme in energy metabolism, and an ATP synthase subunit of *L. vannamei* has been reported to be involved in WSSV infection in by binding to WSSV VP37 [39]. These data implicate that WSSV infection influences the cellular material and energy metabolism in shrimp, and ATP synthase plays a vital role in WSSV infection process.

Arginine kinase was a member of ungrouped proteins and identified from two neighbour spots (spot 11 and 12). Similar results that different spots on a 2D gel are the same protein have been demonstrated in previous proteomic researches [40], [41]. The phenomenon can be explained by protein post-translational modifications, such as glycosylation and phosphorylation, and the modifications can influence p*I*s and MWs of proteins [40]. Arginine kinase is a key enzyme participating in phosphate metabolism [42]. In present study, arginine kinase in hemocytes was up-regulated after WSSV infection at both protein and mRNA level. The data suggest that arginine kinase plays a role in viral infection. Prophenoloxidase, another member of ungrouped proteins, plays a vital role in phenol oxidation reaction, It was down-regulated after WSSV infection at both protein and mRNA level. The down-regulation was also observed in WSSV-infected mud crab through a proteomic method [33]. Some other ungrouped proteins and proteins involved in steroid hormone mediated signal pathway and transmembrane transport were also identified, while the specific functions of most of them in shrimp response to viral infection have not been reported yet, and the subject need further research.

The data in present work was obtained by proteomic approach, while some transcriptomic analyses on shrimp response to WSSV infection have been reported in recent literatures [11], [43], [44]. Our results have some accordance with the findings obtained from these literatures. The molecules identified at both proteomic and transcriptomic levels include SUMO, RNA helicase, dynein, caspase, SOD, heat shock protein 70, phosphatase, alcohol dehydrogenase, actin, tubulin, histone H2B, ATP synthase, arginine kinase and prophenoloxidase. The results suggest that these molecules, especially the immune related proteins, stimulus response proteins and cytoskeletal proteins identified in present work play important roles in shrimp response to WSSV infection.

Quantitative real-time PCR was performed to verify the proteomic data. Although focusing at 24 hpi, the regulated tendencies of the four genes (SUMO, heat shock protein 70, arginine kinase and prophenoloxidase) were consistent with the proteomic data, the protein expression levels and their corresponding mRNA levels did not match perfectly. This phenomenon might be attributed to the complicated and varied post-transcriptional mechanisms involved in turning mRNA into protein [45].

## Supporting Information

Table S1 **Differentially expressed proteins identified by MALDI-TOF-MS or MS/MS in hemocytes of** ***F. chinensis*** **challenged by WSSV.**
(DOC)
