# Supplementary material for: Proteomic Analysis of Differentially Expressed Proteins in Fenneropenaeus chinensis Hemocytes upon White Spot Syndrome Virus Infection
Source: PLoS One. 2014 Feb 27;9(2):e89962. doi: 10.1371/journal.pone.0089962 (PMC3937397; doi:10.1371/journal.pone.0089962)
Supplement: Table S1 — Differentially expressed proteins identified by MALDI-TOF-MS or MS/MS in hemocytes of F. chinensis challenged by WSSV. (DOC) [file pone.0089962.s001.doc]

**Table S1**

| **Spot no.** | **Protein (organism)** | **Accession no.** | **Theoretical p*I*** | **Theoretical MW(Da)** | **Mascot Score /no. of match peptides** | **Protein Coverage (%)** | **Fold change** |
| --- | --- | --- | --- | --- | --- | --- | --- |
| Immune related proteins | | | | | | | |
| 1 | small ubiquitin-like modifier 1 (*Penaeus monodon*) | ACD13593 | 4.99 | 10570 | 82/5 | 32 | 2.03±0.11 |
| 16 | putative RNA helicase (*Marsupenaeus japonicus*) | ABC88642 | 8.91 | 29016 | 52/5 | 29 | 1.84±0.12 |
| 28 | cytoplasmic dynein 2 heavy chain 1 (*Tripneustes gratilla*) | Q27802 | 6.29 | 492912 | 59/71 | 18 | 0.28±0.08 |
| 29 | caspase (*Fenneropenaeus merguiensis*) | AAX77407 | 6.38 | 35667 | 145/7 | 9 | 0.57±0.01 |
| Stimulus response proteins | | | | | | | |
| 4 | cytosolic MnSOD (*Litopenaeus vannamei*) | AAY57407 | 5.62 | 31567 | 59/15 | 9 | 1.60±0.10 |
| 14 | phosphatase 2 (*Culex quinquefasciatus*) | EDS37829 | 5.50 | 54264 | 80/17 | 34 | 2.79±0.10 |
| 19 | heat shock protein 70 (*Pachygrapsus marmoratus*) | ABA02164 | 5.29 | 71556 | 296/4 | 12 | 7.80±0.45 |
| 32 | alcohol dehydrogenase (*Penaeus monodon*) | GO081977 | 6.35 | 45413 | 87/8 | 9 | 0.33±0.11 |
| Proteins involved in glucose metabolic process | | | | | | | |
| 5 | triosephosphate isomerase (*Fenneropenaeus chinensis*) | ABB81879 | 5.55 | 27883 | 77/10 | 60 | 5.47±0.30 |
| 8 | phosphopyruvate hydratase (*Penaeus monodon*) | AAC78141 | 6.18 | 47805 | 127/8 | 9 | 1.68±0.48 |
| 31 | glucose-6-phosphate isomerase (*Alpheus peasei*) | AAK57968 | 6.65 | 17160 | 57/3 | 18 | 0.32±0.12 |
| Cytoskeleton proteins | | | | | | | |
| 7 | tubulin alpha-1 chain (*Homarus americanus*) | Q25008 | 5.00 | 50669 | 83/19 | 49 | 2.19±0.38 |
| 9 | actin (*Drosophila melanogaster*) | AAA28321 | 5.22 | 42147 | 122/4 | 14 | 2.23±0.37 |
| 23 | beta-actin (*Litopenaeus vannamei*) | AAG16253 | 5.30 | 42240 | 220/2 | 25 | 3.81±0.44 |
| 24 | actin A3 (*Bombyx mori*) | CAA28192 | 5.47 | 42207 | 173/4 | 15 | 1.85±0.51 |
| 25 | tubulin beta-1 chain (*Homarus americanus*) | Q25009 | 4.88 | 51167 | 80/15 | 33 | 7.00±0.62 |
| DNA or protein binding proteins | | | | | | | |
| 6 | microtubule-actin cross-linking factor 1, isoform 4 (*Cricetulus griseus*) | EGV92572 | 5.20 | 814017 | 106/83 | 14 | 2.11±0.58 |
| 17 | CAP-Gly domain-containing linker protein 2 (*Rattus norvegicus*) | O55156 | 6.09 | 115990 | 107/27 | 26 | 2.08±0.05 |
| 33 | HMGBb (*Litopenaeus vannamei*) | ADQ43367 | 6.41 | 24208 | 198/6 | 25 | 0.33±0.08 |
| 43 | histone H2B (*Litopenaeus vannamei*) | P83863 | 10.19 | 12767 | 64/12 | 68 | 0.22±0.06 |
| Proteins involved in steroid hormone mediated signal pathway | | | | | | | |
| 15 | nuclear receptor E75 protein (*Fenneropenaeus chinensis*) | ACF36863 | 7.96 | 89185 | 50/10 | 16 | 3.42±0.25 |
| 27 | nuclear progesterone receptor (*Penaeus monodon*) | ADY38993 | 4.39 | 19174 | 264/6 | 22 | 0.32±0.01 |
| ATP synthases | | | | | | | |
| 3 | vacuolar ATP synthase subunit B L form (*Carcinus maenas*) | AAF67183 | 5.31 | 54881 | 62/18 | 33 | 2.46±0.21 |
| 30 | mitochondrial ATP synthase subunit alpha precursor (*Litopenaeus vannamei*) | ADC55251 | 8.97 | 59416 | 53/10 | 20 | 0.41±0.07 |
| Proteins involved in transmembrane transport | | | | | | | |
| 13 | inositol 1,4,5-trisphosphate receptor (*Panulirus argus*) | AAC61691 | 5.92 | 318940 | 80/59 | 22 | 1.92±0.18 |
| 40 | protein transport protein SFT2 (*Ascaris suum*) | ADY43818 | 9.71 | 26975 | 79/9 | 39 | 0.19±0.02 |
| Ungrouped proteins | | | | | | | |
| 11/12 | arginine kinase (*Fenneropenaeus chinensis*) | AAV83993 | 5.92 | 40389 | 72/23 | 51 | 2.24±0.30 |
| 21 | trehalose 6-phosphate synthase 1 (*Callinectes sapidus*) | ACI12944 | 6.92 | 86956 | 56/12 | 20 | 1.81±0.12 |
| 34 | Prophenoloxidase (*Penaeus semisulcatus*) | AAM77690 | 6.24 | 78340 | 62/14 | 17 | 0.53±0.02 |
| 35 | glutamate dehydrogenase (*Litopenaeus vannamei*) | ACC95446 | 6.69 | 61644 | 66/15 | 31 | 0.52±0.11 |
| 38 | Vitellogenin (*Macrobrachium rosenbergii*) | BAB69831 | 9.10 | 287932 | 76/51 | 22 | 0.44±0.14 |
| 39 | unknown protein (*Penaeus monodon*) | GW995866 | 5.74 | 38438 | 84/9 | 13 | 0.64±0.01 |
| 45 | hypothetical protein (*Scylla paramamosain*) | ACY66557 | 5.33 | 17905 | 58/10 | 72 | 0.11±0.06 |
